# Structural Anisotropy-Induced Tensile Stiffening of Fungal Mycelial Networks

**DOI:** 10.1021/acsapm.6c00606

**Published:** 2026-07-06

**Authors:** Stefania Akromah, Neha Chandarana, Stephen J. Eichhorn

**Affiliations:** † Bristol Composites Institute, School of Civil, Aerospace and Design Engineering, 1980 University of Bristol, University Walk, Bristol, BS8 1TR, U.K.; ‡ School of Chemistry, University of Bristol, Cantock’s Close, Bristol, BS8 1TS, U.K.

**Keywords:** Mycelium Films, Fiber Orientation, Unidirectional Composites, Hyphal Alignment, Tensile Properties

## Abstract

We investigate the effect of hyphal orientation on the tensile properties of mycelium films. Hyphal alignment was achieved by exploiting the inherent negative autotropism of fungal growth. Orientation analysis from optical imaging of hyphal apical tips confirmed successful control of directional growth, with unidirectional mycelial films exhibiting a high degree of alignment. The resulting aligned mycelial sheets displayed higher tensile strength, tensile index, and Young’s modulus compared to samples with randomly oriented hyphae. These findings highlight the potential of directional fungal growth control as a biofabrication strategy for tailoring the structure and mechanical anisotropy of mycelium-based materials.

## Graphical abstract

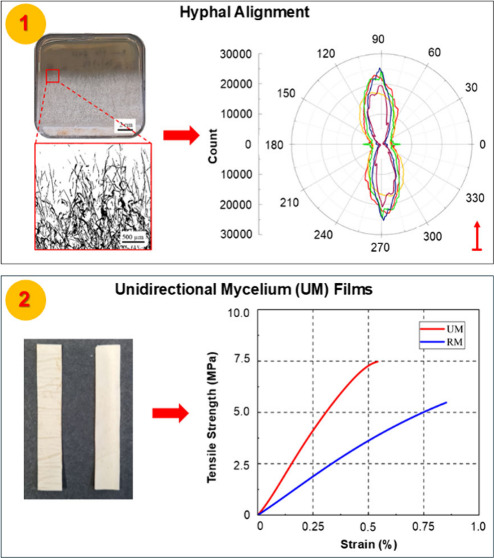

Fungal mycelia are generally characterized by low tensile strength, ranging from ∼0.7 MPa to ∼1.1 MPa for *Pleurotus ostreatus*,^1^ although these values can vary significantly depending on growth conditions, substrate composition, and postprocessing.^2^ This low tensile strength arises from the combined effects of the chemical composition of the fungal cell wall, the strength of interhyphal bonding, and the structural morphology of the hyphal network. Fungal cell walls are primarily composed of nonstructural components, including proteins and lipids, whereas the main structural polymers, chitin and β-glucans, constitute only a minor fraction of the total biomass.^3^ Morphologically, hyphae grow in a disordered, three-dimensional network to maximize substrate colonization and resource acquisition; the uneven distribution and random orientation of hyphal branches create numerous junctions and irregularities that act as stress concentrators.^4^ Moreover, within the networks, individual hyphae are connected mainly through weak secondary interactions and physical entanglement.^5^ Together, these factors result in uneven load distribution and limited tensile mechanical properties, causing premature failure under tension.

It is well-known that the strength and stiffness of synthetic composite materials are strongly influenced by the orientation of their reinforcing fibers, with tensile strength and stiffness maximized when fibers are aligned along the load-bearing direction.^6,7^ This principle is observed not only in engineered composites, such as carbon or glass fiber-reinforced polymers, but also in natural materials like wood, where hierarchical fiber alignment along the growth axis provides enhanced tensile properties. In natural wood, this structural arrangement yields tensile strengths of up to ∼120 MPa along the grain.^8^ However, this strength is not solely due to fiber orientation; it is also supported by the high structural content of cellulose, hemicelluloses, and lignin, as well as strong interfacial bonding between the fibers and the matrix.^9^

We report the effect of the control of hyphal orientation on the tensile strength of mycelium networks. Hyphal alignment was achieved by exploiting the inherent negative autotropism of fungal growth, wherein neighboring hyphae actively avoid contact to optimize spatial expansion and resource utilization. When a single-point inoculum is used, mycelium grows radially, forming a dense but uncontrolled network due to unrestricted lateral branching.^10,11^ In contrast, a linear inoculum strip provides multiple aligned inoculation points, with hyphae extending along the strip avoiding one another, thereby creating physical barriers to lateral branching. As nutrients are consumed along the growth path, a nutrient gradient develops that further encourages forward extension.^10,12^ Together, these physical and chemical cues generate an aligned, unidirectional mycelial network in contrast to the randomly oriented structure obtained from point inoculation.

Mycelia networks were prepared from *Pleurotus eryngii* mycelia grown on malt-extract agar (MEA) media prepared from a solution of 3 wt % malt extract (Formedium), 2 wt % bacteriological agar (VWR), and 400 mL of reverse osmosis water. The MEA solution was sterilized in an autoclave at 121 °C and 15 psi (6.8 × 10^3^ Pa) for 20 min and then poured into 120 mm square Petri dishes to provide a large surface area. Once the MEA media were set, sterile cellophane films (grade: 325P; purchased from AA Packaging Ltd.) were laid on top of their surfaces to subsequently aid in peeling off fully grown mycelia. MEA media were inoculated using a piece of mycelial tissue obtained from a mother culture. Cultures for unidirectional mycelium samples (UM) were prepared by placing 10 × 120 mm mycelial strips along the edge of the MEA medium, as illustrated in Figure 1a­(i). In contrast, cultures for mycelial networks with random hyphal alignment (RM) were inoculated from a single point at the center of the Petri dish to promote radial growth (Figure 1b­(ii)). The inoculated plates were sealed and incubated at 25 °C for 2 weeks. Once fully grown, the mycelial cultures were cut into strips and carefully peeled off the MEA-cellophane backing. The strips were then stacked to form 16-layer samples, sandwiched between two pieces of release film, and hot-pressed at 105 °C for approximately 2 hours until fully dry. Final test specimens were cut from the pressed sheets to dimensions of 6 × 60 mm for tensile testing (Figure S1).

**Figure 1:**
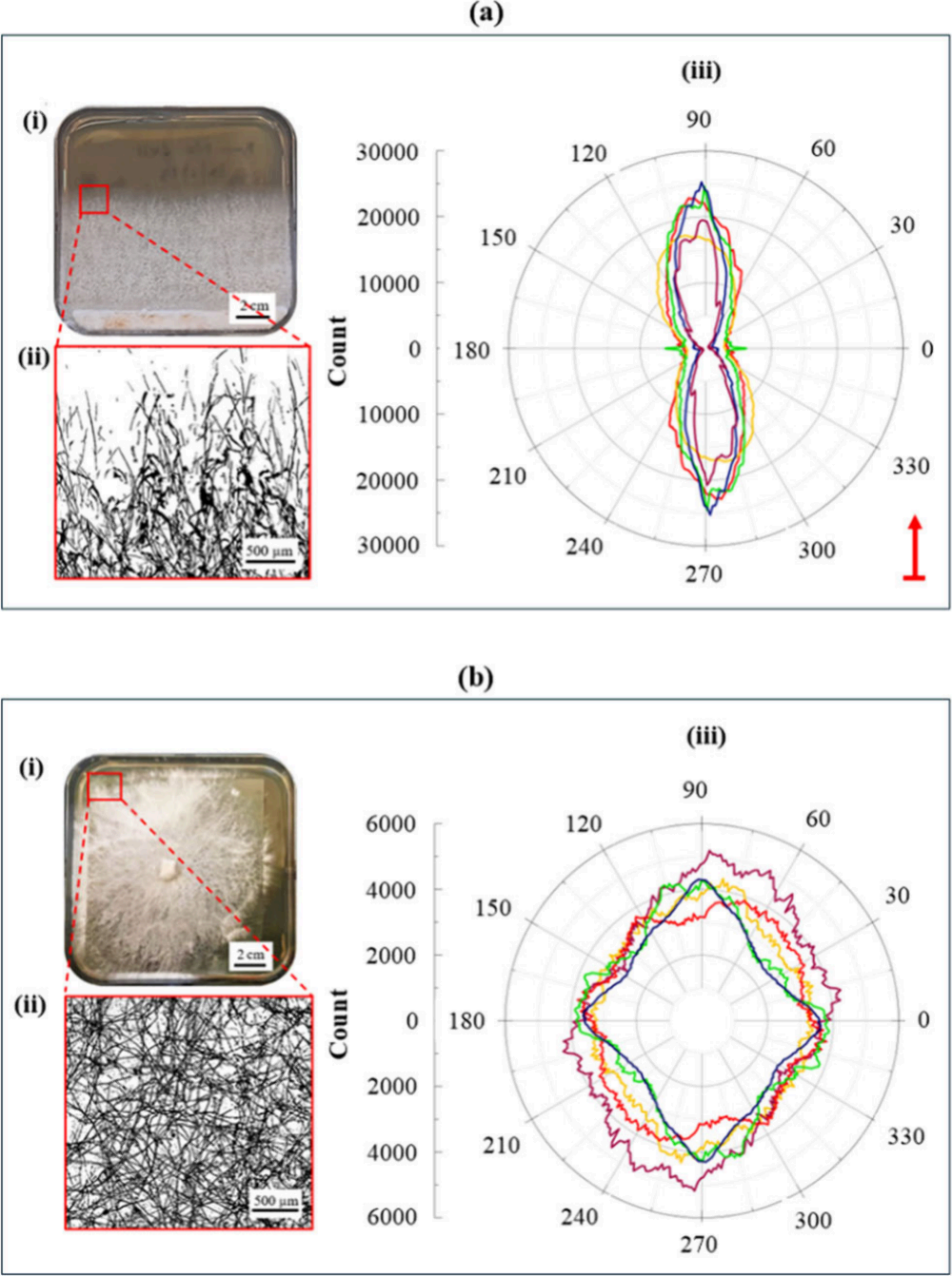
Photographs (i), binarized images of the apical regions obtained by preprocessing optical micrographs in ImageJ (ii), and derived polar (*r, θ*) plots depicting the orientation distributions of hyphal apical tips (iii) for the (a) UM and (b) RM samples. Different colors indicate individual samples. Data illustrate a high degree of directional alignment of the UM samples, in contrast to the isotropic orientation observed for the RM samples.

The morphological properties of as-grown mycelia cultures and mycelia networks were characterized using optical microscopy and a Zeiss Axio Imager M2 microscope. Quasi-static tensile tests were performed by using a Shimadzu testing system with a 1 kN load cell. The ends of the specimens were sandwiched between sandpaper and affixed to the metal grips of the test machine. The crosshead displacement rate was set to 1 mm min^–1^. A noncontact video gauge extensometer was used to measure the elongation of the specimens, with a 30 mm gauge length, in the loading direction.

Figure 1 shows representative photographs, binarized images of the apical regions (obtained by preprocessing optical micrographs in ImageJ), and polar (*r*, θ) data depicting the orientation distributions of the apical tips for the UM and RM samples. While the UM samples (a, i) exhibited pronounced directional alignment (a, iii) along the perpendicular axis, visible also from the image analysis (a, ii) with respect to the inoculum edge, the RM samples (b, i) displayed a near random orientation of the hyphal elements (b, ii), leading to a broad, quasi uniform angular distribution (b, iii), indicative of an isotropic hyphal distribution.

The growth pattern also influenced the areal density, with UM materials exhibiting lower grammages compared to those of the RM samples (Figure 2a). Naturally, mycelia grow through a combination of extension (apical) and branching (apical and lateral), forming a dense, three-dimensional interwoven network that maximizes fungal mass per unit area.^13^ Under forced unidirectional growth, lateral branching is constrained, and hyphae actively avoid overlapping with neighboring filaments due to negative autotropism, resulting in a more sparsely packed network with a lower local density and grammage. The high standard deviation for the values of grammage (Figure 2a) for the RM samples is reflective of the uncontrolled fungal expansion when no restriction is provided, as in the case of UM samples.

**Figure 2:**
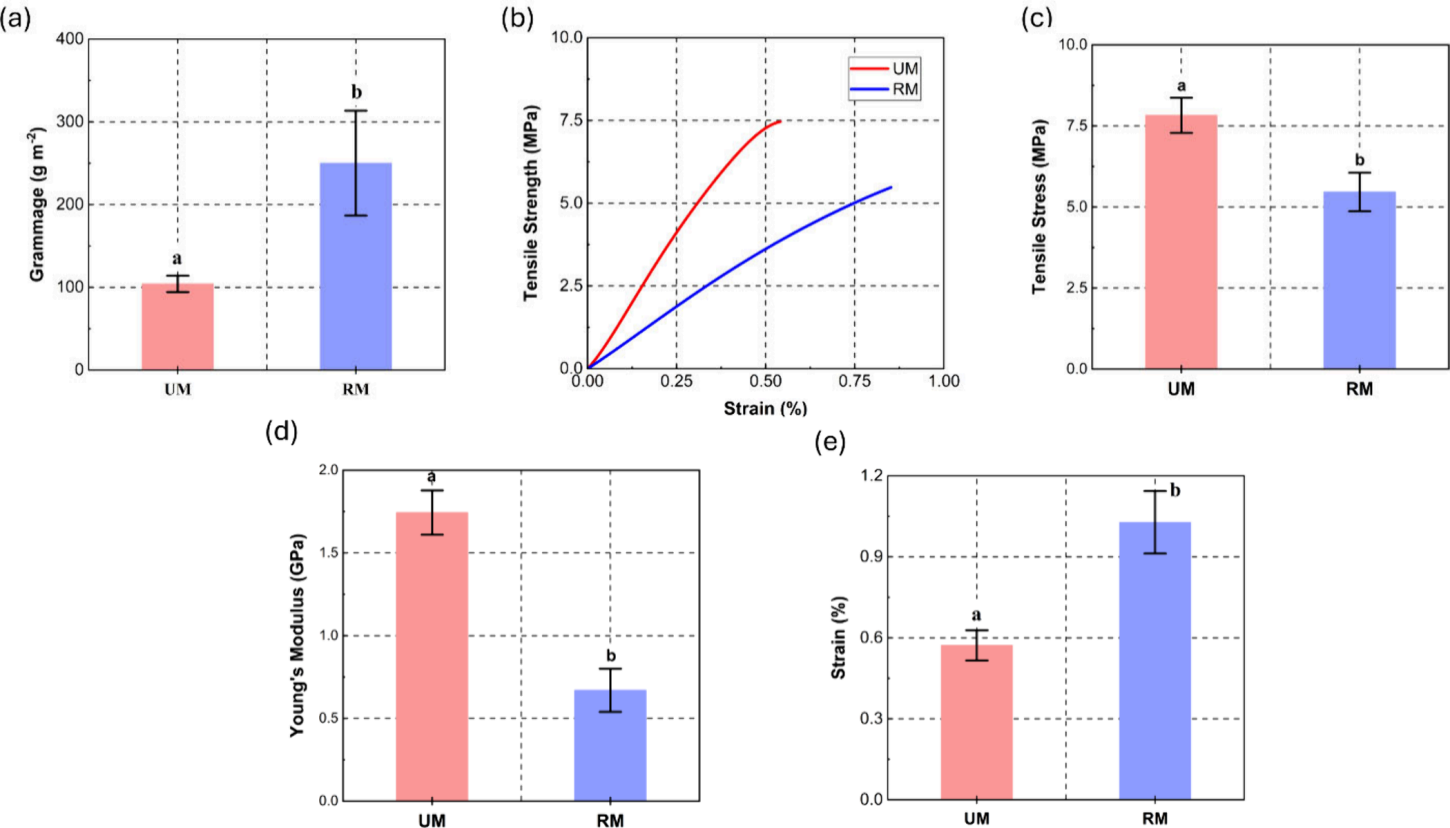
(a) Mean grammage and (b–e) mechanical properties of UM and RM samples. (b) Representative stress–strain curves, (c) tensile stress, (d) Young’s modulus, and (e) strain at failure of UM and RM samples; error bars represent standard deviations (SD). Statistical differences between groups were assessed using one-way ANOVA; distinct letters above the bars indicate statistically significant differences (*p* < 0.05; *n* = 8).

It should be noted that while strip inoculation for UM samples promoted preferential growth direction (Figure 1a), complete hyphal alignment was still not achieved; this is evidenced by the optical micrographs in Figure 1a­(ii) and the spread of the orientation distribution in the corresponding polar plot (Figure 1a­(iii)). Local environmental variations and intrinsic growth dynamics lead to differences in branching and extension rates among individual hyphae, resulting in partial alignment near the inoculum edges and a progressively more heterogeneous, randomly oriented network with increasing distance from the inoculum. This may affect the local mechanical properties of the material, but overall, the general orientation is increased, thereby having, we believe, the overriding influence.

Figure 2b,c reports the tensile mechanical properties of UM and RM samples. Tensile testing of UM samples was carried out parallel to the primary alignment of the hyphal network. As expected, the UM samples exhibited higher tensile strength (Figure 2c) and Young’s modulus (Figure 2d). This is attributed to the structural anisotropy of the mycelial network and the alignment of fungal filaments along the tensile axis. In the UM sheets, hyphae are dominantly aligned parallel to the loading direction, thereby carrying the majority of the applied load, while nonaxial load dissipation is minimal due to limited hyphal branches. This also leads to a more brittle failure of the specimens and, therefore, a lower strain at failure (Figure 2e). In contrast, under uniaxial tension, the randomly oriented hyphae in RM samples allow quasi-isotropic load redistribution, reducing their effective contribution to axial load bearing, thus resulting in lower stiffness and strength. RM samples also display a higher strain at failure (Figure 2e), which is characteristic of randomly oriented fiber composites. Under uniaxial tension, the applied load is redistributed among fibers in multiple orientations, promoting progressive damage and a more gradual, complex failure process.^6,7,14^ Given the change in densities for the UM and RM samples, we also report data in the Supporting Information showing a normalization with respect to (density)^2^, which is typical for porous materials.

Notably, the UM samples exhibited a significantly higher Young’s modulus (*E*_UM_ = 1.74 ± 0.09 GPa) compared to the RM samples (*E*_RM_ = 0.67 ± 0.09 GPa). This difference is consistent with the Krenchel model for fiber orientation effects,^15^ which predicts a relationship of *E*_RM_ = 3/8*E*_UM_ between randomly and unidirectionally oriented fiber composites. Similar differences are noted between the tensile index, which takes into account grammage (g m^–2^), which is often used for paper and similar planar network materials where thickness is negligible; values of 12.51 ± 0.09 N m g^–1^ and 5.61 ± 1.16 N m g^–1^ were obtained for UM and RM samples, respectively (Tables S1 and S2, Supporting Information). Using the Krenchel relationship, the predicted modulus for the UM samples is 1.78 ± 0.23 GPa, in close agreement with the experimental value. It should be noted that the Krenchel orientation factor of 3/8 reflects the effect of fiber alignment for an in-plane, two-dimensional random orientation distribution as assumed in this study. However, this relationship does not account for other factors such as out-of-plane fiber orientation, hyphal branching dynamics, or local network heterogeneity, which also influence network properties.^16^ If these factors were to however influence the stiffness of the sheets, then one would expect to see an effect over and above that observed for orientation. Since this is not the case, it is assumed that orientation of the hyphal networks is the dominant factor governing the mechanical properties. Similar results have been obtained for comparable networks, such as bacterial cellulose,^17,18^ although it has been noted that cross-link density has an effect on these networks, certainly the pellicle properties.^19^

The mycelium networks developed in this study exhibit Young’s moduli comparable to those of a wide range of polymers and natural materials, as shown in the Ashby diagram^20^ of Young’s modulus versus density (Figure 3). With densities in a similar range (see Supporting Information, Tables S1 and S2), these networks occupy a region of the materials property space that is relevant for lightweight structural and semistructural applications. Alignment of the mycelium network leads to a pronounced increase in stiffness, with unidirectionally aligned networks exhibiting higher Young’s moduli than their randomly oriented counterparts, while simultaneously achieving a lower density. This favorable combination of mechanical performance and low mass highlights the potential of mycelium as engineered biobased materials with tunable, orientation-dependent properties. Such characteristics open opportunities for applications including stiff leather-like sheets, thin reinforcement or stiffening layers, and mechanically anisotropic membranes, where directional control of stiffness is desirable.

**Figure 3:**
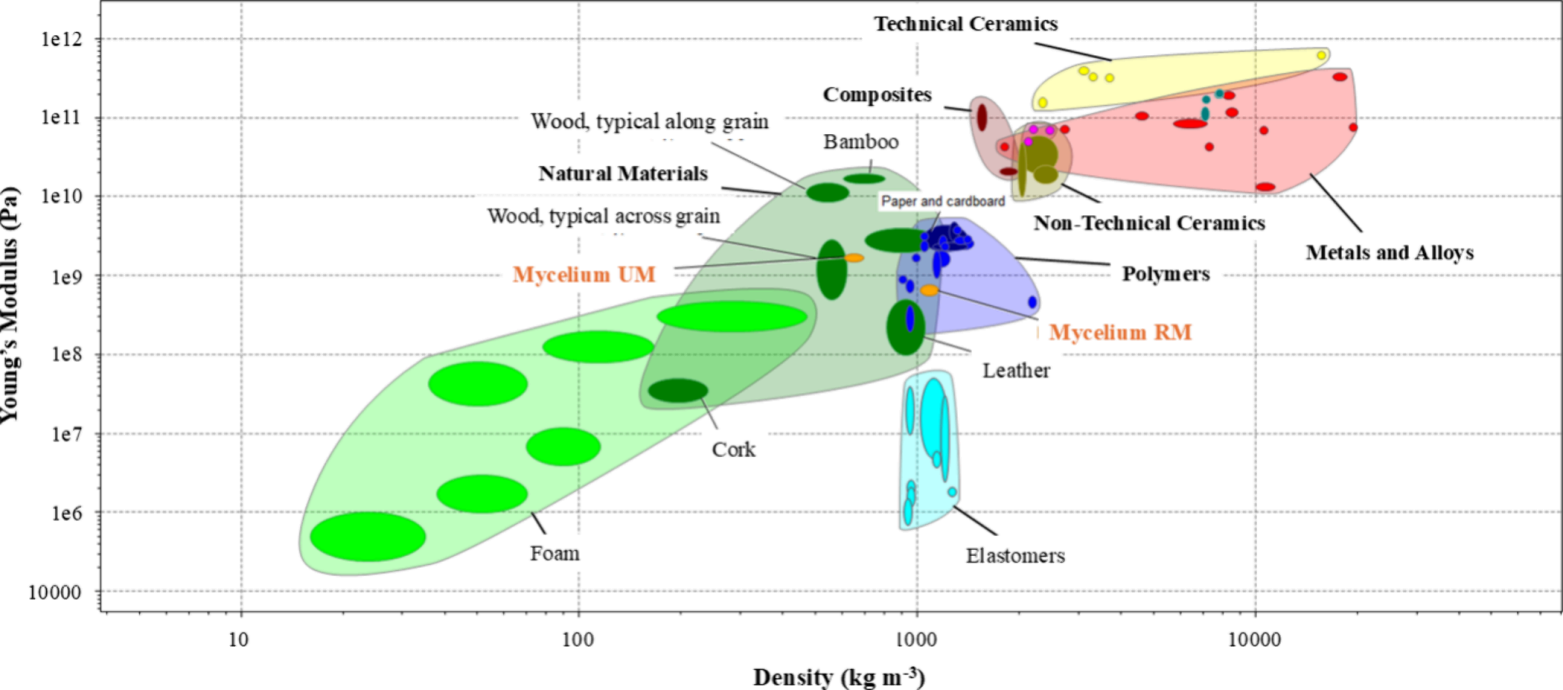
Ashby diagram for a range of materials comparing Young’s modulus versus density on a log-scale. Various natural materials are identified for specific comparison with mycelium materials in this study. Graph and data adapted from Ansys Academic Granta Selector, release 25.2. See Ansys Academic Granta Selector (https://www.ansys.com/products/materials/granta-selector).

This work demonstrates a strategy for controlling mycelial growth to predetermine fiber orientation, thereby enhancing the mechanical properties of mycelium-based materials. By aligning the natural growth of mycelium, it is possible to maximize stiffness while maintaining a low density, similar to how orientation of fibrous structures in other natural materials, such as wood, governs mechanical performance and dimensional stability under variable conditions. While scaling directional growth remains a challenge and further optimization is needed for widespread applicability, this study provides a proof-of-concept for exploiting orientation as a design parameter in engineered mycelium materials. Further work could address the growth characteristics of the networks, controlling cross-link density, as well as fiber orientation to optimize the stiffness in any given direction. The use of this approach could have broad applications in the use of mycelium for civil construction and other materials-based applications where stiffness is a primary design consideration.

## Supplementary Material



## Notes

Table of experimental data: grammage, tensile strength, tensile index, Young’s modulus, and strain at failure (PDF)
