# Supplementary material for: Structural Anisotropy-Induced Tensile Stiffening of Fungal Mycelial Networks
Source: ACS Appl Polym Mater. 2026 Jul 6;8(14):10940–4. doi: 10.1021/acsapm.6c00606 (PMC13410294; doi:10.1021/acsapm.6c00606)
Supplement: Supplementary file 1 [file ap6c00606_si_001.pdf]

**SUPPORTING INFORMATON**

# Structural Anisotropy-Induced Tensile Stiffening of Fungal Mycelial Networks

*Stefania Akromah†*, Neha Chandarana†, Stephen J. Eichhorn†‡**

† Bristol Composites Institute, School of Civil, Aerospace and Design Engineering,

University of Bristol, University Walk, Bristol, BS8 1TR, UK.

‡ School of Chemistry, University of Bristol, Cantock's Close, Bristol, BS8 1TS, UK.

* Stephen J. Eichhorn - † Bristol Composites Institute, School of Civil, Aerospace and Design Engineering, University of Bristol, University Walk, Bristol, BS8 1TR, UK; ‡ School of Chemistry, University of Bristol, Cantock's Close, Bristol, BS8 1TS, UK; orcid.org/0000-0003-4101-273X; Email: s.j.eichhorn@bristol.ac.uk

* Stefania Akromah - Bristol Composites Institute, School of Civil, Aerospace and Design Engineering, University of Bristol, University Walk, Bristol, BS8 1TR, UK; orcid.org/0000-0002-4578-2419; Email: s.akromah@bristol.ac.uk

Neha Chandarana; orcid.org/0000-0002-6361-1533.

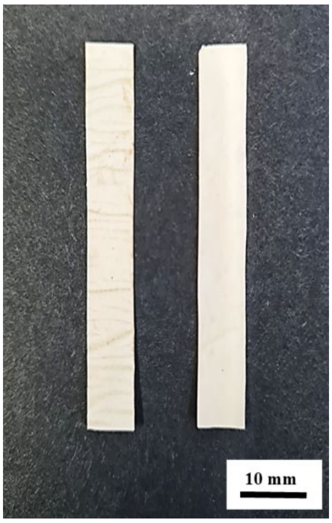


**Figure S1.** Front (left) and back (right) views of a representative mycelium film after hot-pressing. The lines visible on the surface are imprints from the hot-press setup.

(a) (b)

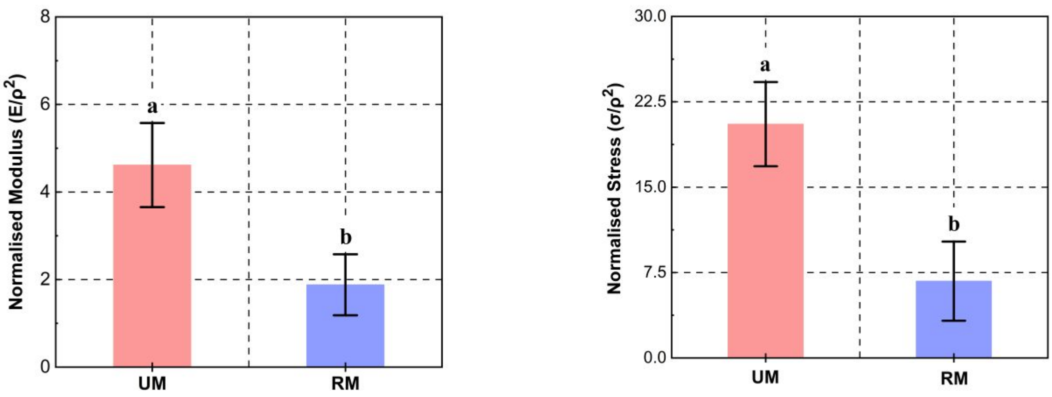


**Figure S2.** Normalised (a) modulus and (b) stress for MC samples (Unidirectional - UM and Random – RM oriented sheets). Data are normalised with respect to (density)$^2$ $(\rho)^2$. A unique letter above the data indicates a statistically significant difference ($p<0.05$; $n=8$).

**Table S1.** Raw data of grammage, tensile strength, tensile index, Young's modulus, and strain at failure of UM samples. SD – standard deviation. Error calculated as $SD/\sqrt{N}$ where N = number of independent measurements

| **Sample #** | **Grammage** | **Tensile Strength** | **Tensile Index** | **Young's Modulus** | **Strain at Failure** | **Density** |
|---|---|---|---|---|---|---|
| **#** | **(g m$^{-2}$)** | **(MPa)** | **(N m g$^{-1}$)** | **(GPa)** | **(%)** | **(g/cm$^3$)** |
| 1 | 111.1 | 7.47 | 11.02 | 1.61 | 0.54 | 0.678 |
| 2 | 111.1 | 8.02 | 12.35 | 1.98 | 0.56 | 0.650 |
| 3 | 111.1 | 7.53 | 10.97 | 2.12 | 0.46 | 0.686 |
| 4 | 83.3 | 7.51 | 13.70 | 2.01 | 0.51 | 0.548 |
| 5 | 83.3 | 6.67 | 14.24 | 1.76 | 0.72 | 0.468 |
| 6 | 138.9 | 6.94 | 7.75 | 1.57 | 0.44 | 0.896 |
| 7 | 111.1 | 8.32 | 12.74 | 1.54 | 0.60 | 0.654 |
| 8 | 83.3 | 10.17 | 17.34 | 1.36 | 0.74 | 0.587 |
| Mean | 104.2 | 7.83 | 12.51 | 1.74 | 0.57 | 0.646 |
| SD | 19.7 | 1.08 | 2.80 | 0.27 | 0.11 | 0.125 |
| Error | 7.0 | 0.38 | 0.99 | 0.09 | 0.04 | 0.044 |

**Table S2.** Raw data of grammage, tensile strength, tensile index, Young's modulus, and strain at failure of RM samples.

| **Sample** | **Grammage** | **Tensile Strength** | **Tensile Index** | **Young's Modulus** | **Strain at Failure** | **Density** |
|---|---|---|---|---|---|---|
| # | **(g m$^{-2}$)** | **(MPa)** | **(N m g$^{-1}$)** | **(GPa)** | **(%)** | **(g/cm$^3$)** |
| 1 | 333.3 | 4.94 | 3.84 | 0.55 | 1.19 | 1.287 |
| 2 | 222.2 | 7.12 | 5.12 | 1.07 | 0.86 | 1.389 |
| 3 | 472.2 | 4.57 | 2.57 | 0.44 | 1.32 | 1.782 |
| 4 | 222.2 | 3.96 | 4.65 | 0.34 | 1.29 | 0.852 |
| 5 | 333.3 | 4.70 | 3.65 | 0.51 | 1.06 | 1.287 |
| 6 | 222.2 | 5.67 | 4.08 | 0.99 | 0.66 | 1.389 |
| 7 | 111.1 | 5.48 | 8.53 | 0.68 | 0.85 | 0.642 |
| 8 | 83.3 | 7.25 | 12.44 | 0.78 | 0.10 | 0.583 |
| **Mean** | 250.0 | 5.46 | 5.61 | 0.67 | 0.92 | 1.151 |
| **SD** | 126.9 | 1.19 | 3.27 | 0.26 | 0.40 | 0.417 |
| **Error** | 44.9 | 0.42 | 1.16 | 0.09 | 0.14 | 0.148 |
